# The Potential Role of Expression of HLA‐E and HLA‐G in Organ Xenotransplantation

**DOI:** 10.1111/xen.70155

**Published:** 2026-08-03

**Authors:** Jeffrey K. Sumbo, Xiaowei Hu, Eckhard Wolf, David K. C. Cooper, Leo H. Buhler

**Affiliations:** ^1^ Faculty of Science and Medicine University of Fribourg Fribourg Switzerland; ^2^ Division of Anesthesiology, Department of Acute Medicine Geneva University Hospitals Geneva Switzerland; ^3^ Gene Center and Department of Veterinary Sciences LMU Munich Munich Germany; ^4^ Center For Innovative Medical Models (CiMM) LMU Munich Oberschleißheim Germany; ^5^ German Center For Diabetes Research (DZD) Neuherberg Germany; ^6^ Center For Transplantation Sciences, Department of Surgery Massachusetts General Hospital, Harvard Medical School Boston Massachusetts USA; ^7^ Surgical Research Unit, Department of Surgery Fribourg Cantonal Hospital, University of Fribourg Fribourg Switzerland

**Keywords:** gene‐editing, HLA‐E, HLA‐G, natural killer cells, pig, xenotransplantation

## Abstract

Xenotransplantation offers a promising solution to the shortage of human organs for transplantation but requires overcoming numerous immune responses—particularly those mediated by the innate immune system through natural killer (NK) cells and macrophages. This review examines advances demonstrating that the expression of human leukocyte antigen E (HLA‐E) on porcine cells contributes to reduce cellular xenograft rejection. HLA‐E expression partially inhibits both direct NK cell cytotoxicity and antibody‐dependent cellular cytotoxicity (ADCC), while also attenuating macrophage‐mediated lysis. Furthermore, perfusion of transgenic porcine organs expressing HLA‐E with human blood resulted in significantly less tissue damage compared to wild‐type counterparts, thereby confirming the protective effect of HLA‐E against innate immunity. Inhibition of NK cell activation can be further enhanced by co‐expression of HLA‐G and HLA‐E. These findings confirm the potential of HLA‐E and HLA‐G expression as a complementary strategy in the design of immune‐compatible porcine organs for clinical xenotransplantation.

**Abbreviations**
ADCC: antibody‐dependent cellular cytotoxicity
B2M: β2‐microglobulin
B4GALNT2/L: β‐1,4‐N‐acetyl‐galactosaminyl transferase 2/‐like
CMAH: cytidine monophosphate‐N‐acetylneuraminic acid hydroxylase
CMV: cytomegalovirus; CRISPR/Cas9, clustered regularly interspaced short palindromic repeats/CRISPR‐associated protein 9
GGTA1: α1,3‐galactosyltransferase
GTKO: α1,3‐galactosyltransferase knockout
HDACi: histone deacetylase inhibitors
hEF1α: human elongation factor 1α
HLA: human leukocyte antigen
ICAM2: intercellular adhesion molecule 2
ILT: mmunoglobulin‐like transcript
ITAM: immunoreceptor tyrosine‐based activation motif
ITIM: immunoreceptor tyrosine‐based inhibitory motif
KIR: killer‐cell immunoglobulin‐like receptor
NK: natural killer (cell)
PAEC: porcine aortic endothelial cells
PBMC: peripheral blood mononuclear cells
pEF1α: porcine elongation factor 1α
SCNT: somatic cell nuclear transfer
SCT: single chain trimer
sgRNA: single guide RNA
SLA: swine leucocyte antigen
TFPI: tissue factor pathway inhibitor
THBD: thrombomodulin
TKO: triple‐knockout.

## 1 Introduction

The limited availability of deceased human donor organs remains a major obstacle to progress in allotransplantation, resulting in long waiting lists. In this context, xenotransplantation, and more specifically the use of gene‐edited pig organs, is emerging as a promising approach, even though it faces significant challenges, to meet this growing demand [1]. Xenografts, like allografts, are confronted with four different types of graft rejection: hyperacute rejection, acute humoral rejection, cellular rejection, and chronic rejection. The inactivation of the gene coding for α1,3‐galactosyltransferase (GTKO) marked a decisive advance, significantly reducing the risk of hyperacute rejection linked to pre‐existing natural anti‐Gal antibodies [2, 3, 4]. Other pig gene deletions and human gene insertions have reduced the incidence of rejection further [5, 6].

However, xenotransplantation does not involve the exact same immunological mechanisms as allotransplantation. Although both processes engage innate and adaptive immunity, innate immune responses play a more prominent role in xenotransplantation than in allotransplantation [7]. Accordingly, whereas T lymphocytes constitute the majority of infiltrating cells during allograft rejection, xenografts predominantly elicit the infiltration of innate immune cells, including NK cells, macrophages, and neutrophils [8].

Yet, despite recent progress, it is possible to further improve pig graft survival by introducing additional genetic modifications. Various complementary approaches are currently being explored, including the expression of immunomodulatory molecules, such as HLA‐E, on porcine cells [9, 10]. HLA‐E, a non‐classical MHC class I molecule composed of the heavy chain of HLA‐E, β2‐microglobulin (B2M) and a peptide derived from the leader sequence of some MHC class I molecules, plays a central role in regulating NK cell responses [10, 11]. These responses are important contributors to cellular rejection [12], either by direct lysis or by antibody‐dependent cell‐mediated cytotoxicity (ADCC) [13, 14], and could therefore help to reduce immune activation and promote graft survival.

The aim of this brief review is to explore the impact of HLA‐E expression on porcine organs intended for xenotransplantation, as an additional strategy for improving immunological compatibility between pig and human and therefore prolonging graft survival. This strategy has been investigated using in vitro models and ex vivo experimental animal models.

## 2 Clinical Evidence From Recent Pig to Human Xenotransplantation Cases

In the recent cases of porcine kidney and heart xenotransplantation in humans, signs of NK cell infiltration and activation have been described and were associated with xenograft rejection. Data collected from the first cases of gene‐edited porcine kidney xenotransplantation in brain‐dead recipients showed increased expression of genes related to NK cell burden within the xenografts, among other signs of antibody‐mediated rejection [15]. More recently, Schmauch et al. [16] described the coordinated adaptive immune response in a brain‐dead recipient receiving a kidney xenotransplant. An increase of blood NK cells between post‐operative day 10–28 was observed, temporally concordant with an expansion of IgA/IgG B cell clonotype, before a biopsy‐confirmed antibody‐mediated rejection at day 33, suggesting that NK cells probably contributed to the early immune response before rejection [16]. During the first compassionate use of porcine cardiac xenotransplantation, single‐cell RNA sequencing also revealed that NK cells were among the most important changes in immune cell type composition across timepoints, along with monocytes, CD4+ T cells, and CD8+ T cells [17]. Based on these clinical observations, an exploration of ways to inhibit NK response in xenotransplantation seems to be necessary.

## 3 Suppression of NK Cell Function by Expression of HLA‐E

**FIGURE 1:**
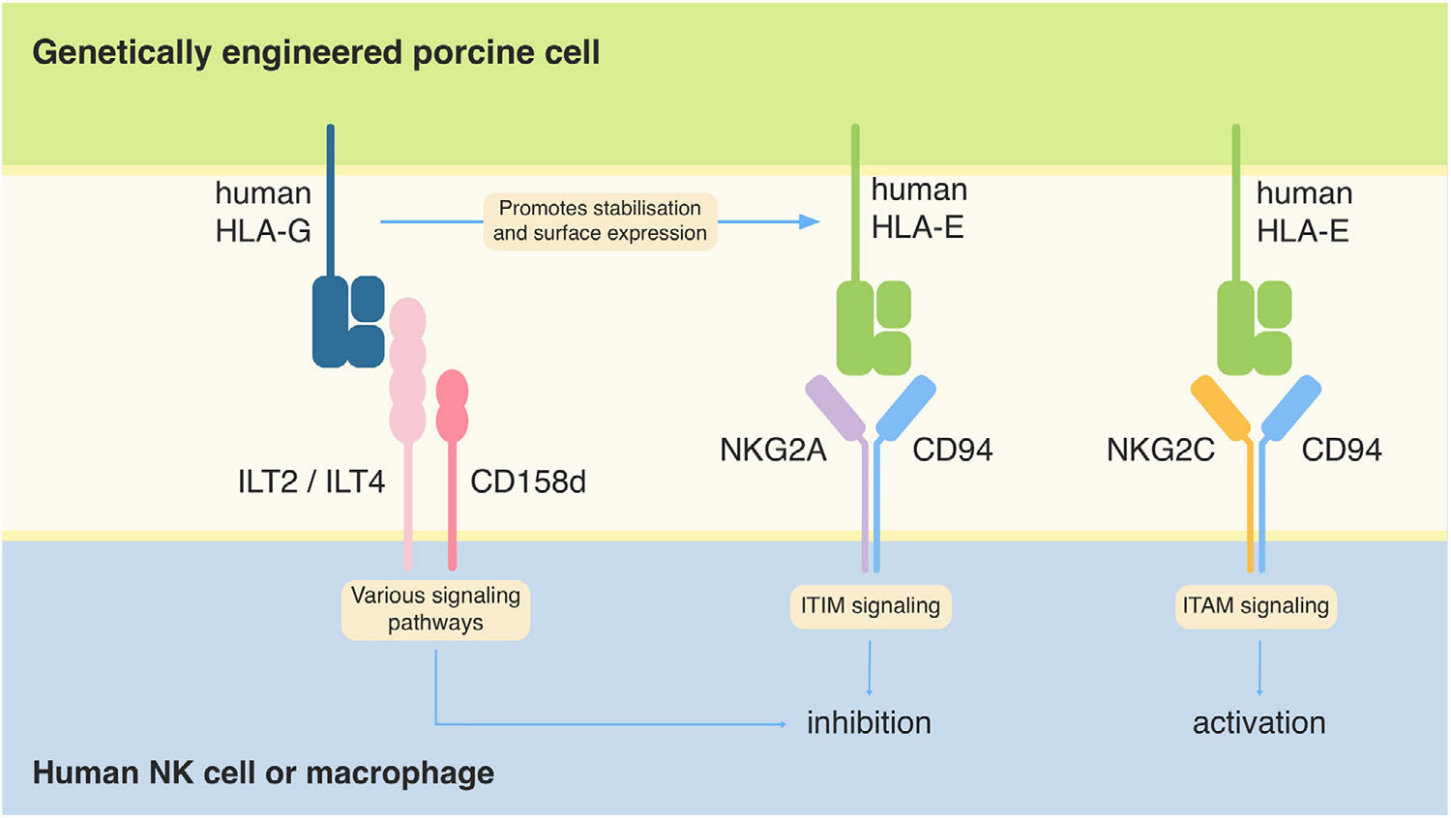
HLA‐E and HLA‐G receptors signaling in natural killer cells and macrophages. Due to its immunoreceptor tyrosine‐based inhibitory motif (ITIM), the CD94/NKG2A complex, that binds HLA‐E, produces inhibitory signals, as do the HLA‐G‐binding immunoglobulin‐like transcript 2 (ILT2), ILT4 and CD158d through various signaling pathways. On the other hand, the HLA‐E‐binding CD94/NKG2C complex, which contains an immunoreceptor tyrosine‐based activation motif (ITAM), produces an activating signal. HLA‐G further promotes immunotolerance through cleavage of its leader sequence, which produces a peptide that promotes stabilization and surface expression of HLA‐E.

NK cell function is regulated by a variety of activating or inhibiting surface receptors, such as killer‐cell immunoglobulin‐like receptor (KIR) or NKG2A [11]. CD94 is a member of the killer cell lectin‐like receptors (KLR) that can dimerize with various NKG2 proteins, including NKG2A and NKG2C, consequently forming HLA‐E‐recognizing receptors [18]. For instance, the CD94/NKG2A complex, which contains an immunoreceptor tyrosine‐based inhibitory motif (ITIM), produces an *inhibitory* signal [11, 18]. Conversely, the CD94/NKG2C heterodimer contains an immunoreceptor tyrosine‐based activation motif (ITAM), that makes it an HLA‐E‐specific *activating* receptor [11, 18] (Figure 1). Despite sharing high sequence similarity, NKG2A/CD94 binds HLA‐E with a much higher affinity (6 to 10 times) than NKG2C, regardless of which peptide is bound to HLA‐E. This difference in affinity appears to arise from structural variations within the dimer, which influence the manner in which CD94 presents its contact interface to HLA‐E [10, 19, 20]. It should be emphasized that NK cell activity is regulated by a wide range of interactions, and that the receptors mentioned above do not constitute an exhaustive list [11].

Human NK cells differentiate between “self” and “non‐self”. The presence of self MHC class I molecules on autologous cells is a potent inhibitory signal that suppresses NK cytotoxicity [9]. However, human NK cells poorly recognize porcine MHC molecules, and are therefore not inhibited, which explains the NK cytotoxicity observed in xenotransplantation [9, 21]. Expression of HLA‐E on pig cells, recognized by the CD94/NKG2A complex, might compensate for this deficiency and therefore reduce NK cytotoxicity [21]. Kwiatkowski et al. have shown that upregulation of SLA class I expression on porcine endothelial cells, induced by TNF‐α treatment, lead to a reduction in human NK cell‐mediated lysis, thereby suggesting the presence of a certain degree of functional interaction [22]. However, the reduction in lysis associated with SLA was not observed at basal levels of expression; moreover, in contrast to HLA‐E, SLA elicited a lymphocyte‐mediated response that was detrimental to the cells [21, 22].

NK cells do not act solely through direct cytotoxicity; they can also be recruited by antibodies in a process known as antibody‐dependent cellular cytotoxicity (ADCC) [23, 24]. NK cell antibody‐dependent cellular cytotoxicity is mediated by the binding of the Fc‐receptor CD16 with antibodies that are bound to the target cell [25]. The CD94/NKG2A inhibitory complex nevertheless directly interferes with CD16‐mediated signaling, including the CD16‐induced Fas/FasL pathway [26], which prevents downstream activation in allogeneic contexts [23]. Furthermore, the degree of HLA‐E‐mediated inhibition depends on the peptide presented by HLA‐E. Rölle et al. [27] showed that distinct HLA‐E peptide complexes differentially modify antibody‐driven effector functions of adaptive NK cells. Most xenotransplantation studies, however, measure overall NK cytotoxicity or degranulation without specifically separating direct cytotoxicity from ADCC [28]. Future studies using antibody‐depleted blood or blocking Fc receptors would help clarify HLA‐E's specific impact on ADCC in xenotransplantation.

In human allotransplantation, upregulated expression of HLA‐E has been associated with acute cellular rejection of renal allografts and correlates with a higher incidence of HLA class I leader (signal) peptide mismatches, increased infiltration of T cells and NK cells, and reduced graft survival [29]. In the context of mismatched donor‐derived leader peptides, HLA‐E may function as an alloantigen, potentially recognized by recipient effector cells. This mismatch can alter the peptide–HLA‐E complex, shifting receptor engagement from the inhibitory CD94/NKG2A pathway toward the activating NKG2C receptor on NK cells, thereby promoting graft rejection [30]. Furthermore, there is emerging evidence that IgG or IgM antibodies may recognize the HLA‐E–leader peptide complex, contributing to antibody‐dependent NK cell‐mediated cytotoxicity [31].

Human NK cells are not the only responsive cells to HLA‐E expression: Maeda et al. demonstrated that pig cells expressing HLA‐E are also protected against macrophage‐mediated cytotoxicity, another contributor to cellular rejection, by the same mechanism that inhibits NK cells, namely HLA‐E‐CD94/NKG2A binding [32].

## 4 Expression of HLA‐E in Pigs

Expression of HLA‐E in pigs has been explored by different research groups, including Weiss et al. [33], who first generated transgenic pigs expressing HLA‐E and human B2M. In a 7.7‐kb genomic *HLA‐E* fragment exon 1 was replaced by exon 1 of *HLA‐B7*, since the HLA‐B7 signal sequence‐derived peptide (VMAPRTVLL) has the strongest effect on HLA‐E cell surface expression [33]. The resulting *HLA‐E* ^sigB7^ fragment was coinjected with a 15‐kb genomic human *B2M* fragment into pronuclei of zygotes (landrace hybrid background) [33]. The founder animal with the highest number of integrated copies and the best transgene expression was used for establishing a line [33]. The transgene inheritance pattern suggested co‐integration of the *HLA‐E* ^sigB7^ and *B2M* fragments at a single genomic locus [33]. High‐level membrane expression of HLA‐E/B2M was reported in PBMCs and endothelial cells of various organs, including heart, kidney, pancreas, lung liver and brain [33]. Notably, the expression of HLA‐E was higher in the transgenic pig cells than in cell lines naturally expressing HLA‐E/B2M, arguing against an adverse effect of HLA‐E overexpression in vivo [33]. More recently, Yue et al. [34] demonstrated the feasibility of engineering genetically multi‐modified pigs (knockout of 3 pig genes, insertion of 9 human transgenes including HLA‐E/B2M). Porcine skin fibroblasts were modified in a single step by co‐transfection with: i) expression plasmids for Cas9 and *GGTA1*‐, *CMAH*‐ and *B4GALNT2*/*B4GALNT2L*‐specific gRNAs; ii) a plasmid encoding PiggyBac transposase; and iii) a large PiggyBac vector containing three multi‐gene expression cassettes (hCD46/hCD55/hCD59 driven by a hEF1α promoter, hHLA‐E/hB2M fusion protein/hCD47 driven by the CAG promoter, and hTHBD/hTFPI/hCD39 driven by an ICAM2 promoter) [34]. The genes within each cassette were linked by 2A “ribosome‐skip” signals [34]. A triple‐knockout (TKO) cell clone with an insertion of the 9‐gene PiggyBac vector in one *GGTA1* allele was used for somatic cell nuclear transfer (SCNT) to generate founder animals [34]. Expression studies of umbilical vein endothelial cells from the genetically modified pigs showed similar expression levels as for the corresponding genes in human umbilical vein endothelial cells for all transgenes except for hTHBD [34]. Their biological functionality was demonstrated with a range of in vitro assays, addressing human antibody binding, protection against complement or NK cell cytotoxicity, phagocytosis, and modulation of coagulation [34].

Amino acid substitutions can enhance the cell‐surface expression of HLA‐E [35]. Matsuura et al. [35] showed that the amino acid at position 147 plays a key role in HLA‐E expression on the cell surface. They found that substituting the serine at this position with cysteine, thereby producing the variant named HLA‐Ev(147), markedly increased its surface expression in both human and pig cells [36]. By producing a transgenic mouse expressing the HLA‐Ev(147) gene, they further demonstrated that this gene could be expressed in animals [35]. Samiec et al. [37] demonstrated that HLA‐E expression could also be increased in triple‐transgenic adult cutaneous fibroblast cells by epigenetic modifications using trichostatin. A, a non‐specific inhibitor of histone deacetylases (HDACi) that enables epigenetic reprogramming of cell transcriptional and translational activity.

The choice of promoter is important in regard to the expression of the molecule. Indeed, the use of promoters such as those from human α‐globin and human eukaryotic translation elongation factor 1α (hEF1α) results in uneven expression levels of target genes [38]. Conversely, the CAG promoter (composed of elements from cytomegalovirus and the chicken β‐actin promoter) induced high expression of target genes in genetically modified pigs [38]. Sun et al. [38] identified for the first time a novel function of a sequence within the porcine elongation factor 1α (pEF1α) gene that may act as a xenogeneic serum‐inducible promoter. They showed that promoters such as pEF1α and CAG enable similar HLA‐E expression in porcine kidney‐15 (PK‐15) cells. Under basic culture conditions in porcine aortic endothelial cells (PAECs), the pEF1α promoter induced significantly lower HLA‐E expression than the CAG promoter. However, it achieved higher HLA‐E expression than CAG in PAECs when cultured in primate serum‐supplemented culture medium, showing an upregulation of gene expression in response to certain xenogeneic stimuli [38]. Yet, the activity of the promoters was not affected by porcine serum [38]. Furthermore, the CAG promoter undergoes methylation‐mediated transcriptional silencing over time, whereas the activity of the pEF1α promoter was sustained at the initial level during long‐term culture, and might therefore be preferred in the context of xenotransplantation [38].

## 5 In Vitro Studies

Forte et al. [10] analyzed the effect of HLA‐E expression on cell lysis of porcine endothelial cells and lymphoblastoid cells by human NK cells. They obtained a reduction of 24%–44% of human NK cell‐mediated cytotoxicity against porcine lymphoblastoid cells [10]. In the presence of blocking anti‐HLA class I, anti‐CD94 and anti‐NKG2A monoclonal antibodies, the previously observed inhibition of cytotoxicity against pig cells induced by HLA‐E expression was consistently reversed. NK cells regained their ability to kill pig cells, indicating a direct inhibitory effect of transgenic HLA‐E expression on xenogeneic anti‐pig NK cytotoxicity [10, 14, 33]. Although HLA‐E expression led to a decrease in cytotoxicity, it did not fully suppress it. The partial nature of the reduction in cytotoxicity is most likely explained, at least partially, by the presence of NKG2A‐negative subpopulations in polyclonal NK populations [10, 33].

These findings were corroborated by Lilienfeld et al. [14], who also assessed the reduction in NK‐mediated cytotoxicity caused by HLA‐E expression on both porcine bone marrow–derived cells and porcine aortic endothelial cells. These cells expressed a HLA‐E single chain trimer (SCT) composed of a HLA‐E binding peptide antigen, the mature human B2M and the mature HLA‐E heavy chain [14]. The average reductions in human NK‐mediated cytotoxicity were 51% in porcine endothelial cells and 49% in porcine aortic endothelial cells [14].

Weiss et al. [33] reported that the level of CD94/NKG2A expression on the human NK cells correlated with the reduction of cytotoxicity on the target cells. Relative lysis, that is the proportion of HLA‐E‐positive target cells destroyed compared to HLA‐E‐negative controls, was measured at 35% when CD94/NKG2A expression on NK cells was high, 62% when it was intermediate, and 72% when it was low.

## 6 The Combination of Expression of HLA‐E and G

Increased expression of HLA‐G in human *allografts* has been frequently associated with improved graft acceptance in solid organ transplantation, primarily through the modulation and downregulation of the host immune response [39]. As a molecule involved in the regulation of NK cell activity, its potential use in xenotransplantation has also been studied [12]. Its expression on porcine cells alone without other non‐classical HLA class I molecules has shown intermediate inhibitory effects on human NK cell‐mediated lysis [40, 41], possibly in a dose‐dependent way [42]. However, transgenic expression of HLA‐G alone might not be sufficient to prevent human NK cell activity on transgenic porcine cells, as its protective effect is only partial and depends on inhibitory receptor (immunoglobulin‐like transcript 2; ILT‐2) expression that is inconsistent across NK cell lines [43]. In this context, its co‐expression with HLA‐E may lead to a synergistic suppression of NK cell‐mediated immune responses in xenotransplantation. Furthermore, HLA‐G (in its G1 form) not only inhibits the NK cell‐mediated immune response but also reduces macrophage‐mediated cytotoxicity, through binding to ILT2, ILT4, and CD158d [42, 44] (Figure 1). Rao et al. [45] have even demonstrated in vitro suppressive effect of HLA‐G1 expression in GTKO porcine fibroblasts on both human and monkey T, B and NK cell. The *HLA‐G1* sequence (codon optimized for the expression in pigs) was inserted by CRISPR/Cas9‐mediated homology‐directed repair into exon 1 of the porcine *ROSA26* locus of Mangalitsa pig fetal fibroblasts [45]. A moderate level of transgene expression driven by the *ROSA26* promoter was intended to avoid potential negative effects of strong overexpression. Pigs produced from these cells by SCNT revealed consistent HLA‐G1 expression in association with the endogenous porcine B2M in heart, lungs, liver, kidneys and pancreatic islets [45]. Notably, HLA‐G1 expression had no effect on the ability of transplanted islets to normalize glycemia of immune‐deficient diabetic mice [45].

The co‐expression of HLA‐G with HLA‐E has been shown to further enhance the inhibition of NK cell activation in the xenogeneic context. HLA‐G indirectly supports HLA‐E function by serving as a source of leader peptides, much like classical MHC class I molecules. The cleavage of HLA‐G's leader sequence produces a peptide that binds to the groove of HLA‐E and promotes its stabilization and surface expression. This complex, in turn, engages the inhibitory receptor CD94/NKG2A with high affinity [46]. This mechanism is physiologically relevant in immune‐privileged environments such as the placenta, where HLA‐E and HLA‐G are co‐expressed to contribute to maternal‐fetal immune tolerance. Supporting this, Cross‐Najafi et al. demonstrated that porcine endothelial cells engineered to express both HLA‐E and HLA‐G showed a significantly greater reduction in NK cell activation compared to cells expressing either molecule alone [30]. Evidence also suggests that combined HLA‐E and HLA‐G expression may have immunosuppressive effects through CD94/NKG2‐independent pathways [47].

## 7 Ex Vivo Experimental Perfusion Models

Abicht et al. [48] tested the effect of the expression of HLA‐E by perfusing genetically‐modified (GTKO/hCD46/HLA‐E/hB2M) porcine hearts ex vivo with human blood. After subjecting the heart to 150 min of ischemia at 4°C, they perfused it for a total of 8 h at 36°C–37°C. The genetically‐modified hearts showed superior myocardial perfusion, cardiac indices and stroke volume, and less edema formation compared to wild‐type organs [48].

Similarly, Laird et al. [49] perfused genetically‐modified (GTKO/hCD46/HLA‐E/hB2M) pig lungs with human blood for 4 h or until failure. All five pig lungs remained viable throughout the 4‐h perfusion period, whereas the GTKO/hCD46 control group lungs showed an average survival time of only 162 min, with higher histamine elaboration and platelet activation, probably related to greater endothelial dysfunction. Pulmonary vascular resistance was also significantly higher during the first 3 h of perfusion in the control group [49].

These findings correlated with those of Puga Yung et al. [21] who showed a reduced and slightly delayed recruitment of NK cells during perfusion of HLA‐E/hCD46 double‐transgenic porcine limbs, as well as partially reduced NK cytotoxicity, as demonstrated by the 64.3% reduction in porcine endothelial cell lysis compared to wild‐type limbs. While this reduction was significant when compared to perfusion of wild‐type pig limbs, the perfusion of limbs from hCD46 single‐transgenic pigs did not show any reduction in cell lysis in vitro [21].

In a similar vein, Bongoni et al. [50, 51] studied the influence of both hCD46 and HLA‐E expression on complement‐mediated xenograft rejection by perfusing forelimbs of wild‐type and hCD46/HLA‐E double‐transgenic pigs ex vivo with either heparinized human blood or autologous pig blood. Although a significant decrease in (i) endothelial activation, (ii) tissue apoptosis, and (iii) coagulation as well as (iv) reduced C6 deposition was observed in the transgenic pig limbs, the absence of studies in either hCD46 or HLA‐E single‐transgenic pigs makes interpretation of the effect of HLA‐E on these parameters uncertain [50, 51]. In vitro studies showed no difference in the extent of complement activation between hCD46 and hCD46/HLA‐E pig cells, suggesting a minor role for HLA‐E in this process [50, 51]. Nevertheless, it remains possible that HLA‐E contributed to the reduction of apoptosis by reducing NK cell reactivity, as NK cells are known to have the capacity to induce apoptotic cell death in myocytes [50].

## 8 In Vivo Studies

These are limited, but of some relevance to the effect of HLA‐E expression on the outcome of a xenograft is the study by Watanabe et al. [52]. These authors investigated whether a transgenic intra‐bone bone marrow transplant could elicit sufficient chimerism to improve the survival of a porcine lung xenograft in a non‐human primate. hCD46/HLA‐E GalTKO bone marrow transplantation proved insufficient to induce stable chimerism or significantly improve survival of lung transplants in baboons [52].

## 9 Discussion

Multiple experiments highlight the promising role of expression of HLA‐E as a strategy to enhance the immunological compatibility of porcine xenografts. They demonstrate that HLA‐E expression on porcine endothelial cells significantly reduces human NK cell‐induced cytotoxicity but, unlike classical MHC class I molecules, does not elicit an allogeneic T‐cell response [14, 33], making it a particularly attractive immunomodulatory molecule for xenotransplantation.

However, in order for HLA‐E to achieve the desired effect, its expression on pig cells must be stable and sufficient. In vitro studies have shown that achieving this level of expression in porcine endothelial cells necessitates extrinsic pulsing with HLA‐E binding peptides and/or IFN‐γ stimulation, which is not conceivable in a living organism [10]. To overcome this limitation, the use and expression of single chain trimers, as suggested by Lilienfeld et al., could be a promising approach, as it ensures constitutive and physiologically relevant expression of HLA‐E on the cell surface without relying on exogenous peptides [14].

As the surface expression of HLA‐E is highly dependent on the availability of these appropriate leader peptides, the co‐expression of HLA‐G is another strategy to overcome this limitation by serving as a natural source of high‐affinity peptide for the stabilization of HLA‐E, as suggested by both Crew [53] and Cross‐Najafi et al. [30].

It is also essential to acknowledge that while HLA‐E inhibits NK cells through interaction with the CD94/NKG2A inhibitory receptor, it can also bind to the CD94/NKG2C activating receptor, potentially promoting immune activation [33]. Nevertheless, experimental evidence shows that this proinflammatory pathway is normally counteracted by the inhibitory pathway, suggesting that the net effect of HLA‐E expression in the xenotransplantation context is inhibitory, resulting in reduced cytotoxicity [33, 49]. However, this balance may be disrupted under certain conditions, since different individuals may exhibit different NK cell subsets varying in their NKG2A/NKG2C expression. Patients infected with cytomegalovirus, for example, exhibit a dramatic proliferation of NKG2C^high^ NK cell subsets lacking NKG2A expression [54], which persist for more than a year after the acute infection [55]. The proportion of NK cells expressing CD94/NKG2A receptors can therefore be subject to important inter‐individual differences, which can vary between 20% and 80% of the NK cell population in different human subjects at different timings [53], once again underscoring the importance of combinatorial approaches. Indeed, HLA‐G engages ILT2, which is expressed on a subset of these NKG2C^high^ cells, and may therefore contribute to mitigating their immune‐activating effects [56].

Depletion of NKG2C^high^ cells would not be beneficial, as this subset, paradoxically, plays a protective role against CMV infection. Eliminating these cells would increase the risk of CMV disease, a major cause of morbidity and graft loss in transplantation [57].

## 10 Conclusion

In conclusion, HLA‐E expression in gene‐edited porcine organs offers a method for mitigating innate immune responses, especially those mediated by human NK cells and macrophages, and represents a possible approach to improving survival after xenotransplantation. The co‐expression of HLA‐G shows potential to enhance cell surface expression of HLA‐E and broaden the efficacy of HLA‐E‐mediated NK cell inhibition in xenografts, which should also be explored, along with the impact and effects of various levels of expression of HLA‐E and HLA‐G.

## Author Contributions

Jeffrey K. Sumbo and Xiaowei Hu contributed equally to the study. David K. C. Cooper and Leo H. Buhler designed the study; Jeffrey K. Sumbo, Xiaowei Hu and Eckhard Wolf collected the data; Jeffrey K. Sumbo, Xiaowei Hu, Eckhard Wolf and Leo H. Buhler interpreted the data; Jeffrey K. Sumbo, Xiaowei Hu, Eckhard Wolf and Leo H. Buhler drafted the manuscript. David K. C. Cooper participated in the writing of the paper and its critical analysis.

## Conflicts of Interest

David K. C. Cooper is a consultant to eGenesis Bio, Cambridge, MA, but the opinions expressed in this article are those of the authors and do not necessarily represent those of eGenesis. Leo H. Buhler is a consultant to Clonorgan Biotechnology Co., Ltd., but the opinions expressed in this article are those of the authors and do not necessarily represent those of Clonorgan. The other authors report no conflict of interest.
